# Methotrexate Failure in the Treatment of Adult-Onset Still’s Disease: A Case Report

**DOI:** 10.7759/cureus.27283

**Published:** 2022-07-26

**Authors:** Kameron Tavakolian, Mihir Odak, Steven Douedi, Viraaj Pannu, Swapnil V Patel

**Affiliations:** 1 Department of Internal Medicine, Jersey Shore University Medical Center, Neptune City, USA

**Keywords:** biologic treatment, systemic steroids, anakinra, oral methotrexate, adult onset still's disease (aosd)

## Abstract

Adult-onset Still's disease (AOSD) is a rheumatological condition associated with significant morbidity and mortality. Typically a diagnosis of exclusion, the therapeutic management has relied mainly on symptom control and immune suppression. Methotrexate (MTX), a disease-modifying anti-rheumatoid drug (DMARDs), has become a drug of choice in treating several autoimmune conditions, including AOSD. Unfortunately, despite being largely effective, this medication can result in treatment failure, exacerbation, and a flare of symptoms. We present the case of a 31-year-old male who presented to us with weakness and palpitations, who was ultimately found to have a flare of his Still's disease, despite being on MTX therapy. Our hope is to encourage a suspicion for treatment failure in patients with similar symptoms, in order to encourage a faster initiation of alternative therapies to alleviate their discomfort.

## Introduction

Adult-onset Still's disease (AOSD) is an uncommon condition, with an incidence of 0.16 cases per 100,000 people. It is characterized by quotidian fevers, arthritis, and a salmon-colored evanescent rash most commonly seen on the trunk. In addition, patients are often found to have elevated serum ferritin levels and non-specific signs of elevated acute phase reactants [1]. Although AOSD is typically treated with methotrexate (MTX), which has been recognized as a well-tolerated medication, particularly with joint involvement, therapy success rates remain to be seen with regard to AOSD. Here, we present a case of an acute flare of AOSD due to a failure of MTX therapy [2]. We hope to encourage a high index of suspicion for MTX failure in AOSD flares to initiate appropriate therapeutics early to reduce patient morbidity and mortality.

## Case presentation

**Figure 1:**
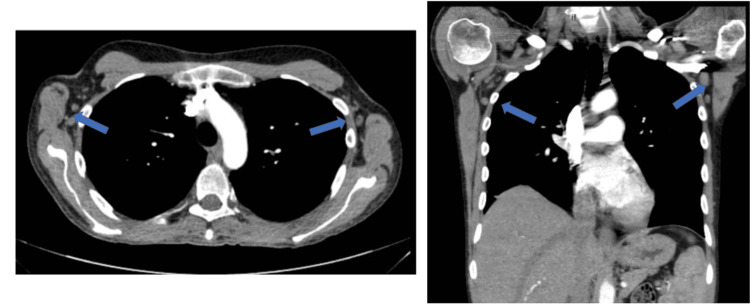
CTA of chest demonstrating bilateral axillary lymphadenopathy (blue arrows). CTA: Computed tomography angiography.

**Figure 2:**
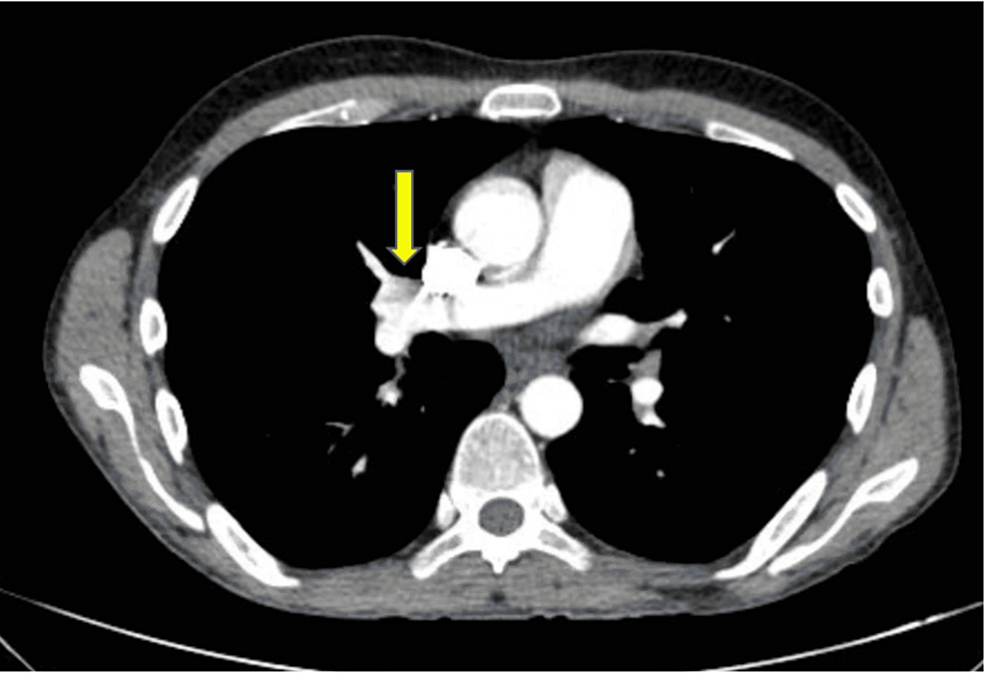
CTA of chest demonstrating right hilar lymphadenopathy (yellow arrow). CTA: Computed tomography angiography.

**Figure 3:**
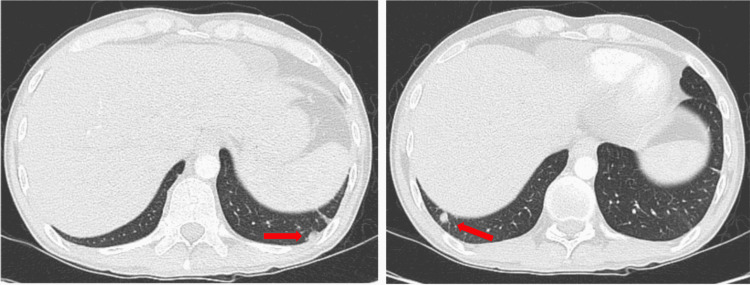
CTA of chest demonstrating multiple pulmonary nodules (red arrows). CTA: Computed tomography angiography.

A 31-year-old male with a past medical history significant for AOSD diagnosed one year prior, presented to the ED with weakness and palpitations. He reported daily fevers, with a peak temperature of 104.6 degrees Fahrenheit and intermittent joint pains. The patient also had been experiencing palpitations while changes in body position and showering. He noted he ran out of his oral prednisone 20 mg daily maintenance therapy several weeks prior and failed to take oral MTX therapy due to GI side effects and persistent fevers and joint pain. He denied chest pain, dyspnea, headache, nausea, vomiting, or skin changes. Upon arrival, his vitals were temperature 98.2 ℉, blood pressure 132/84 mmHg, heart rate 98 beats per minute, and saturating 100% on room air. Physical exam revealed swelling and decreased range of motion at bilateral shoulder, elbow, wrist, metacarpophalangeal, proximal interphalangeal, knee, ankle, and ​​metatarsophalangeal joints. Laboratory data was notable for a WBC count of 21.3 x103/uL (normal value: 4.5-11.0x103/uL), hemoglobin of 8.9 g/dL (normal value: 12.0-16.0 g/dL), and platelet count of 798x103/uL (normal value: 140-450x103/uL). He was also noted to have an elevated serum ferritin of 1139 ng/mL (normal value: 24-336 ng/mL) and C-reactive peptide of 21.98 mg/dL (normal value: 0.00-0.74 mg/dL) as shown in Table 1. Antinuclear antibody and rheumatoid factor were both negative. Computed tomography with angiography (CTA) of the chest was negative for a pulmonary embolism. However, it demonstrated bilateral axillary (Figure 1) and right hilar lymphadenopathy (Figure 2) and multiple pulmonary nodules, with the largest measuring 9 millimeters (Figure 3). An ECG was not performed.

**Table 1:** Initial laboratory results.

Serum	Results	Reference range
WBC (x10^3^/uL)	21.3	4.5-11.0 (x10^3^/uL)
Hemoglobin (g/dL)	8.9	12.0-16.0 (g/dL)
Platelet count (x10^3^/uL)	798	140-450 (x10^3^/uL)
Serum ferritin	1139	24-336 ng/mL
C-reactive peptide	21.98	0.00-0.74 mg/dL
Antinuclear antibody	Negative	Negative
Rheumatoid factor	Negative	Negative

The patient was started on IV Solu-Medrol 60 mg every 12 hours and transitioned to oral prednisone 40 mg daily after a significant improvement in symptoms was observed following 24 hours of IV steroids. Prior to discharge, the patient was instructed to follow up at our rheumatology clinic with the plan of starting a biologic agent at that time since he had failed MTX therapy. Despite close adherence to medical therapy, however, his symptoms had not abated at his one-month follow-up appointment. At that point, he was restarted on high-dose prednisone (50 mg daily) and was transitioned to Anakinra due to MTX failure.

## Discussion

AOSD is a systemic inflammatory disorder. Although the etiology is unknown, it is thought that a combination of genetic susceptibility and infectious triggers predispose patients to develop AOSD [3]. Patients suspected to have AOSD classically present with a triad of quotidian fever, arthritis, and evanescent rash. Some other common features include myalgias, hepatosplenomegaly, lymphadenopathy, sore throat, pleurisy, weight loss, abdominal pain, pleurisy, and pericarditis [4]. Based on two epidemiological studies in France and Japan, the estimated incidence ranges from 0.16 to 0.4 in 10,000 people [5,6]. There is an equal distribution among males and females and a bimodal peak between ages 15-25 and 36-46 years [7]. One five-year retrospective study determined the mortality rate for AOSD to be 2.6% in hospitalized patients in the United States between the years 2009 and 2013 [8]. A prospective study several years earlier by Ruscitti P et al. reported a mortality rate of 16% [9]. This discrepancy may be attributed to the fact that the patients in the Ruscitti P et al. study were referred to tertiary care specialist centers, suggesting a greater disease severity. The feared complication of AOSD is macrophage activation syndrome (MAS), which is seen in up to 17% of cases and carries a mortality rate as high as 42% [10,11]. Characteristic laboratory findings of AOSD include elevated erythrocyte sedimentation rate (ESR), C-reactive protein (CRP), leukocytosis with neutrophilic predominance, anemia, thrombocytosis, and elevated serum ferritin [12]. Additionally, ferritin levels are typically higher in AOSD than are classically seen in other autoimmune, inflammatory, and infectious disorders [13]. The combination of elevated ferritin and a glycosylated ferritin level ≤20% demonstrated a sensitivity and specificity for AOSD of 70.5% and 82.3%, respectively, in a retrospective multicenter of 205 patients [14]. Specificity increased to 92.9% when combined with ferritin levels greater than five times the upper limit of normal [14]. Our patient's laboratory results yielded an undetectably high ferritin level at the time of his diagnosis and a level of 1,139 ng/mL on his subsequent admission. Various diagnostic criteria have been proposed for the diagnosis of AOSD, including Yamaguchi's, Cush's, and Calabro's criteria. These criteria evaluate patients based on a combination of symptoms and lab values [15]. The fourth criterion from Fautrel was proposed, which utilized glycosylated ferritin with similar sensitivity and improved specificity [16]. However, Fautrel's criteria may have limited utility since the measurement of glycosylated ferritin is not readily available in all healthcare facilities [17]. Diagnostic testing may include complete blood count, comprehensive metabolic panel, inflammatory markers, blood cultures, chest X-ray, abdominal ultrasound, CT and/or fluorodeoxyglucose (FDG)-positron emission tomography (PET) scans, and bone marrow or lymph node biopsies. Before diagnosis, our patient had multiple hospital admissions where numerous diagnostic imaging tests of different modalities were conducted. In addition, an extensive rheumatological and infectious disease workup was unremarkable, including and not limited to, antinuclear antibodies, double-stranded DNA antibodies, cyclic citrullinated peptide antibodies, ribonucleic protein antibodies, rheumatoid factor, complement levels, and human leukocyte antigen-B27.

For new-onset disease, nonsteroidal anti-inflammatory drugs (NSAIDs) can be used for supportive treatment during diagnostic workup [3]. Corticosteroids and MTX are the mainstay of treatment and have proven to be most effective in treating AOSD [15]. Biologic agents have an essential role in chronic disease and steroid-dependent patients. These agents include tumor necrosis factor (TNF)-alpha inhibitors, interleukin-1 receptor antagonists, and interleukin-6 antagonists [15]. IV immune globulin is a controversial therapeutic option that has been used in patients who are refractory to or have contraindications to steroid therapy [16]. AOSD is a diagnosis of exclusion, and therefore, the path taken leading up to the diagnosis can be costly and time-consuming. Of the potential biomarkers, ferritin and glycosylated ferritin are most promising and may have a role in diagnosis, assessing disease activity, prognosis, and treatment. Further study into effective and efficient diagnostic approaches for AOSD is warranted.

## Conclusions

AOSD can be challenging to diagnose and treat. While expert consensus exists that aids in our management strategy, alternate strategies would have included the use of high-dose IV corticosteroids in addition to cyclophosphamide. As there have been reported cases of disease-modifying anti-rheumatoid drugs (DMARDs) failures in the literature, analysis of these therapies and further research into the development of alternative treatments are warranted. Further study is therefore warranted into the long-term outcomes of the use of biologic therapy and IVIg.
